# *Staphylococcus aureus* Causing Tropical Pyomyositis, Amazon Basin, Peru 

**DOI:** 10.3201/eid1901.120819

**Published:** 2013-01

**Authors:** Coralith García, Marie Hallin, Ariane Deplano, Olivier Denis, Moises Sihuincha, Rozanne de Groot, Eduardo Gotuzzo, Jan Jacobs

**Affiliations:** Author affiliations: Universidad Peruana Cayetano Heredia, Lima, Peru (C. García, E. Gotuzzo);; Université Libre de Bruxelles, Brussels, Belgium (M. Hallin, A. Deplano, O. Denis);; Hospital Iquitos Cesar Garayar García, Iquitos, Peru (M. Sihuincha);; Universiteit Leiden, Leiden, the Netherlands (R. De Groot);; Institute of Tropical Medicine Antwerp, Antwerp, Belgium (J. Jacobs)

**Keywords:** tropical pyomyositis, Staphylococcus aureus, S. aureus, Panton-Valentine leukocidin, Peru, Amazon Basin, methicillin-susceptible Staphylococcus aureus, MSSA, community-associated methicillin-resistant S. aureus clones, MRSA, bacteria, staphylococci

## Abstract

We studied 12 *Staphylococcus aureus* isolates causing tropical pyomyositis in the Amazon Basin of Peru. All isolates were methicillin-susceptible; 11 carried Panton-Valentine leukocidin–encoding genes, and 5 belonged to multilocus sequence type 25 and possessed an extensive set of enterotoxins. Our findings suggest sequence type 25 is circulating in tropical areas of South America.

Pyomyositis is an acute bacterial infection characterized by suppuration within large skeletal muscles manifesting as single or multiple abscesses. Its exact pathogenesis is unknown but is thought to occur through bacteremic seeding (*1*). The infection is often seen in tropical countries, hence the name tropical pyomyositis, and usually occurs in young, otherwise healthy persons. In addition, the infection is increasingly reported from temperate regions in patients receiving immunosuppressive therapy or with HIV infection (*1*,*2*). The most common bacterial causes of tropical pyomyositis are *Staphylococcus aureus* (90% in tropical areas, 75% in temperate zones) and group A streptococcus (1%–5%); less common causes are group B, C, and G streptococcus, pneumococcus, *Haemophilus* spp., and gram-negative bacilli (*2*). Furthermore, the increasing incidence of pyomyositis in temperate regions has been correlated with the emergence and spread of community-associated (CA)–methicillin-resistant *S. aureus* (MRSA) clones (*3*). These CA*-*MRSA clones usually produce Panton-Valentine leukocidin (PVL), a pore-forming toxin encoded by 2 genes, *lukF-PV* and *lukS-PV* (*4*).

Few studies have been done on the genetic characteristics of *S. aureus* that cause tropical pyomyositis. To help fill this void, we determined the molecular characteristics of *S. aureus* isolates causing tropical pyomyositis in the Amazon Basin of Peru.

## The Study

We analyzed 12 *S. aureus* isolates from patients with tropical pyomyositis. Of the 12 isolates, 10 were obtained from patients hospitalized in a 120-bed public hospital located in Iquitos, the largest city in the Amazon Basin of Peru (160,000 inhabitants). A retrospective chart review showed that 38 patients were hospitalized with tropical pyomyositis during 2009–2010; these patients represented 0.9% of 4,445 hospital admissions. The 10 isolates from hospitalized patients came from these patients; the 2 other isolates were obtained in 2005 from 2 persons hospitalized in Yurimaguas, a city 388 km southeast of Iquitos (63,000 inhabitants). We obtained clinical data by reviewing patients’ charts.

Isolates were analyzed at the Instituto de Medicina Tropical Alexander von Humboldt in Lima, Peru. Colonies were identified as *S. aureus* by Gram staining and by positive reactions for catalase, DNAse, and tube coagulase tests. We screened for oxacillin resistance by using the cefoxitin (30 μg) disk diffusion test, and we used disk diffusion to assess susceptibilities to clindamycin, erythromycin, gentamicin, ciprofloxacin, rifampin, and trimethoprim-sulphamethoxazole (*5*). 

Detection of 16S rRNA, *mec*A, and *nuc* genes by multiplex PCR was performed at the Centre National de Référence *S. aureus*, Erasme Hospital, Brussels, Belgium (*6*). We used PCR to detect the presence of the PVL- and toxic shock syndrome toxin 1–encoding genes (*luk*S-*luk*F PV and *tst*, respectively), exfoliatin A– and B–encoding genes (*eta* and *etb*, respectively) (*7*), and enterotoxin genes (*sea, seb, sec, sed, see, seg, seh, sei, sej, sek, sem, sen, seo, sep, seq, ser*) as described by Omoe et al. (*8*). We determined *spa* type as described (*9*), and we performed multilocus sequence typing on 1 randomly selected isolate of each *spa* type (*10*).

Demographic characteristics of the patients are shown in the Table. Of the 12 patients, 9 (75%) were <5 years of age, similar to age distributions recorded in other studies (*2*). No patient revealed a history of chronic disease. Two groups of muscles were involved in 2 patients, and 10 patients had fever. All patients had local edema and pain, needed surgical drainage, and had received antimicrobial drugs (oxacillin and/or clindamycin) before samples were obtained.

**Table:** Characteristics of patients with tropical pyomyositis caused by methicillin-susceptible *Staphylococcus aureus*, Amazon Basin, Peru*

Patient age	Year patient hospitalized	Body site involved	*S. aureus* isolate							
			*spa* type	Multilocus ST†	Gene					Enterotoxin genes
					*mec*A	*eta*	*etb*	*luk*S-*luk*F	*tst*	
12 mo	2005	Lower limb	t6465	**ST121**	−	−	−	+	−	*sei*, *seg*
3 y	2005	Lower limb	t6465	ST121	−	−	−	+	−	*sei*, *seg*
6 mo	2009	Upper limb	t701	**ST6**	−	−	−	+	−	*sea*
12 y	2009	Thorax, lower limb	t078	**ST25**	−	−	−	+	−	*seb*, *sed*, *sei*, *sej*, *seg*, *sem*, *sen*, *seo*, *ser*
24 mo	2009	Lower limb	t078	ST25	−	−	−	+	−	*seb*, *sed*, *sei*, *sej*, *seg*, *sem*, *sen*, *seo*, *ser*
45 y	2009	Thorax	t1778	**ST1**	−	−	−	+	−	*seh*
3 y	2009	Abdomen, upper limb	t701	ST6	−	−	−	+	−	*sea*
28 d	2010	Lumbar	t164	**ST20**	−	−	−	-	−	*seg*, *sei*, *sem*, *sen*, *seo*
3 y	2010	Lower limb	t078	ST25	−	−	−	+	−	*seb*, *sed*, *sei*, *sej*, *seg*, *sem*, *sen*, *seo*, *ser*
26 y	2010	Abdomen	t078	ST25	−	−	−	+	−	*seb*, *sed*, *sei*, *sej*, *seg*, *sem*, *sen*, *seo*, *ser*
4 y	2010	ND	t1778	ST1	−	−	−	+	−	*seh*
18 mo	2010	Thorax	t078	ST25	−	−	−	+	−	*seb*, *sed*, *sei*, *sej*, *seg*, *sem, sen*, *seo, ser*

*ST, sequence type; −, negative; +, positive; ND, no data. †**Boldface** indicates isolates that were randomly selected by *spa* type for multilocus sequence typing (MLST). The other isolates were not analyzed by MLST. Results for those isolates were extrapolated from results for the analyzed isolates of the same *spa* type.

Of the 12 *S. aureus* isolates we studied, 11 (92%) carried PVL genes; PVL genes were also reported in 12% of clinical MSSA isolates recovered in hospitals in Lima (*11*). In the United States and France, PVL has been detected in 20% and 10%, respectively, of CA-MSSA soft tissue infections (*3*,*12*). *S. aureus* carrying PVL has received more attention by public health officials since the emergence, in the United States and other countries, of skin and soft tissue infections caused by the PVL-positive CA-MRSA clone USA300. A recent publication from the United States noted that 69% of MRSA isolates causing skin and soft tissue infections carried PVL (*3*).

None of the isolates described in this study carried genes associated with the toxic shock syndrome toxin (*tst*) or the staphylococcal scalded skin syndrome (*eta*, *etb*), but all possessed several enterotoxin genes. The presence of toxin genes was strongly linked to *spa* type (Table). In particular, isolates belonging to *spa* type t078 (sequence type [ST] 25) harbored the *egc* operon (coding genes *seg*, *sei*, *sem*, *sen*, *seo*), coding genes *sed, sej* and *ser* (probably carried on the same plasmid) (*13*), and *seb.* Along with toxic shock syndrome toxin 1, *seb* is one of the most powerful staphylococcal superantigens.

Of particular interest in our study is the high proportion of isolates belonging to ST25. This ST has been detected in bovine and human isolates in the United Kingdom (*14*), and an ST25 PVL–positive MSSA was detected in a patient in Brazil with life-threatening sepsis with pneumonia and myositis (*15*). ST25 PVL–positive isolates are thought to be rare in Peru: in 2008–2009, only 6 (3.6%) of 169 MSSA blood isolates from hospitalized patients in Lima (non-tropical area of Peru) harbored *spa* type t078 or related *spa* types (C. Garcia, unpub. data). Our findings raise the hypothesis that ST25 is circulating in the tropical areas of South America.

This study has several limitations. First, tropical pyomyositis cases from the referring hospitals were defined by the patients’ physician, and isolates from all patients were not assessed. Second, because this was a retrospective study, many variables were not recorded, including history of blunt trauma and duration of antimicrobial drug therapy. However, given that studies of the genetic characteristics of *S. aureus* that cause tropical pyomyositis are lacking and given the striking presence of PVL genes among the isolates in our study, our findings add to the insights about the pathogenesis of this acute suppurative infection.

## Conclusions

This study describes a high rate of PVL encoding genes among *S. aureus* causing tropical pyomyositis in the Amazon Basin of Peru. Further investigation in areas geographically different from the Amazon Basin should be done to confirm the association of PVL and other toxins in the pathogenesis of tropical pyomyositis.
